# Supplementary material for: 27-Hydroxylation of oncosterone by CYP27A1 switches its activity from pro-tumor to anti-tumor
Source: J Lipid Res. 2023 Nov 20;64(12):100479. doi: 10.1016/j.jlr.2023.100479 (PMC10770617; doi:10.1016/j.jlr.2023.100479)
Supplement: Supplemental data [file mmc1.docx]

**Supplementary Material**

**27-hydroxylation of oncosterone by CYP27A1 switches its activity from pro-tumor to anti-tumor**

Silia Ayadi^1,2,3^, Silvia Friedrichs^4^, Regis Soulès^1,2,3^, Laly Pucheu^1,2,3^, Dieter Lütjohann^4^, Sandrine Silvente-Poirot^1^, Marc Poirot^1,2,3#^ and Philippe de Medina^1,2,3#^

*^1^ Cancer Research Center of Toulouse (CRCT), Inserm, CNRS, University of Toulouse, Team INOV:"Cholesterol Metabolism and Therapeutic Innovations”, Toulouse, France.^2^ Equipe labellisée par la Ligue Nationale contre le Cancer, Toulouse, France.^3^ French network for Nutrition physical Acitivity And Cancer Research (NACRe network), Jouy en Josas, France.^4^ Institute of Clinical Chemistry and Clinical Pharmacology, University Hospital Bonn, Bonn, Germany.*

Supplementary data S1

Lipid maps codes for new compounds

LMST01010580 | 27-hydroxy-OCDO

LMST01010579 | 25-hydroxy-OCDO

LMST01010581 | 25-hydroxy-cholestanetriol

LMST01010582 | 27-hydroxy-cholestanetriol

LMST01010583 | 25-hydroxy-5,6beta-epoxycholesterol

LMST01010584 | 25-hydroxy-5,6alpha-epoxycholesterol

LMST01010585 | 27-hydroxy-5,6alpha-epoxycholesterol

LMST01010586 | 27-hydroxy-5,6beta-epoxycholesterol

**Supplementary Table 1**

Expression in mRNA of enzymes. Data are takeen from the Human Protein Atlas (https://www.proteinatlas.org/)

| mRNA (T.P.M.) | MCF7 | MDA-MB231 | MDA-MB468 | HepG2 |
| --- | --- | --- | --- | --- |
| CYP27A1 | 0.0 | 5.1 | 39.4 | 217.7 |
| CH25H | 0.0 | 1.2 | 0.0 | 0.0 |
| CYP46A1 | 0.3 | 0.0 | 0.0 | 0.0 |

**Supplementary figure S1**. Mass spectrum of compound I corresponding to the TMS derivative of 27H-5,6ß-EC at the average retention time of 28.07 to 28.35 min.


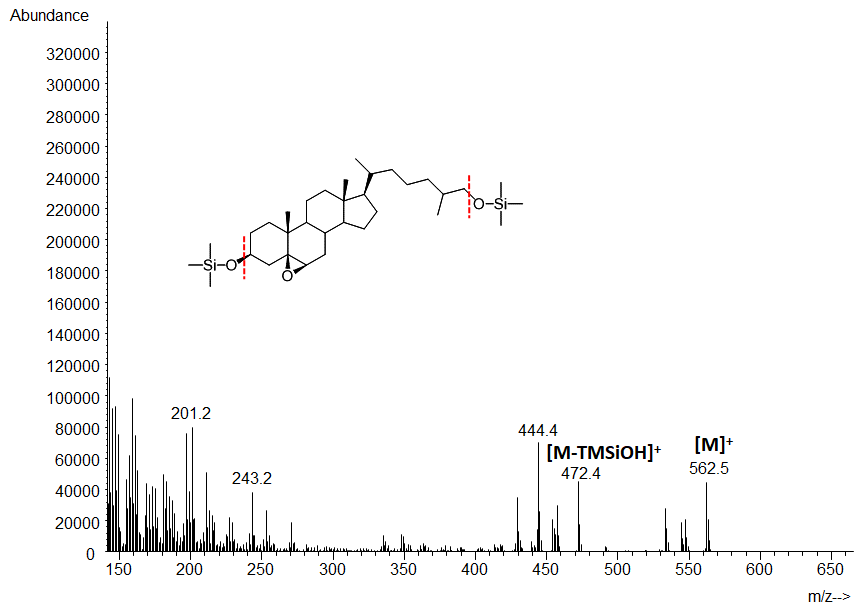


**Supplementary Figure S2:** Mass spectrum of compound II corresponding to the TMS derivative of 27H-5,6α-EC at the average retention time of 29.17 to 29.81 min.


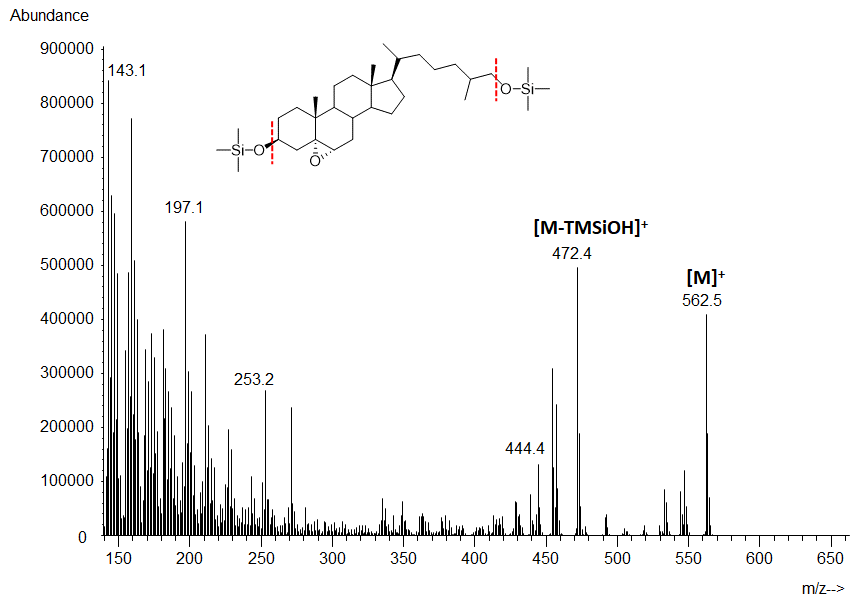


**Supplementary Figure S3:** Mass spectrum of compound III corresponding to the TMS derivative of 27H-OCDO at the average retention time of 31.27 to 31.73 min.


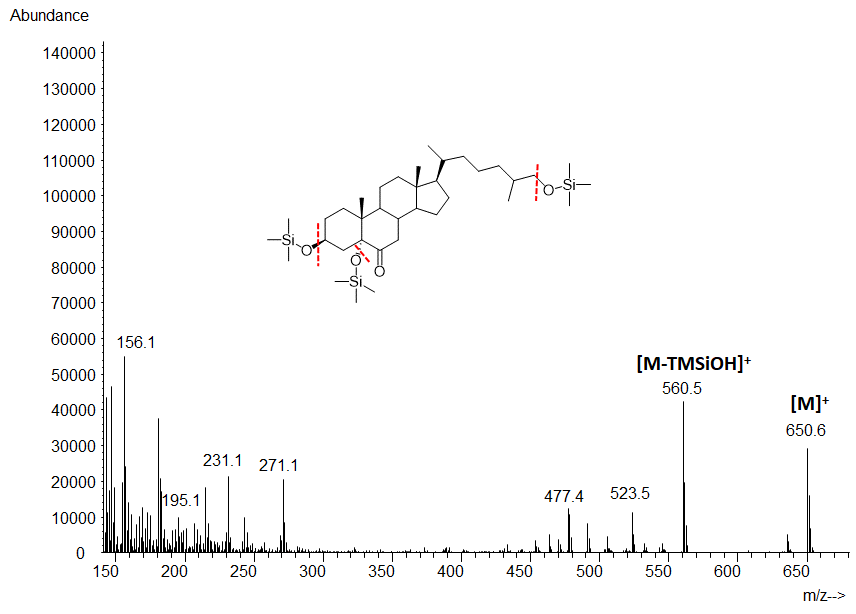


**Supplementary Figure S4:** Mass spectrum of compound IV corresponding to the TMS derivative of 27H-CT at the average retention time of 31.75 to 32.41 min.


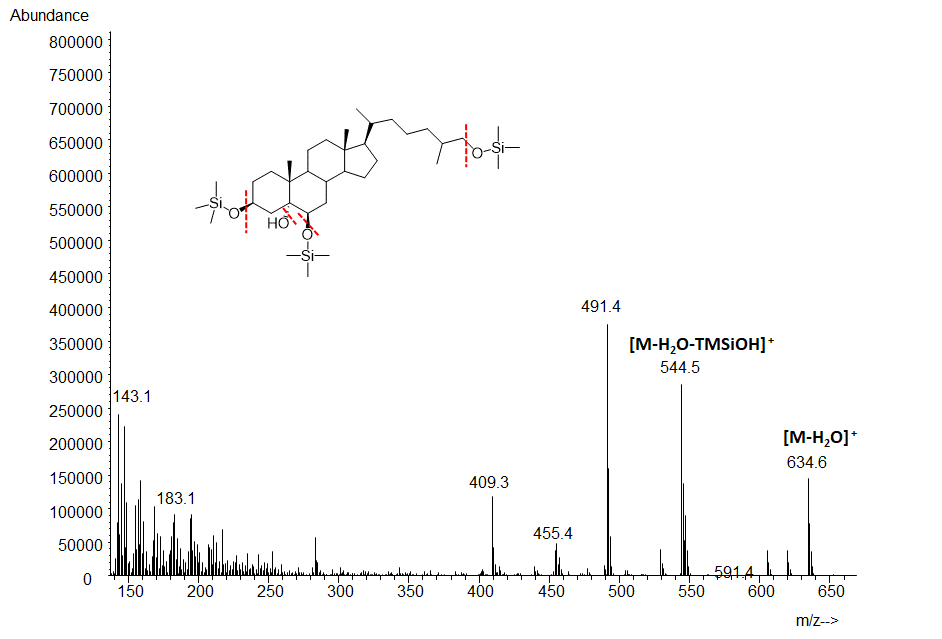


**Supplementary Figure S5**: Total ion chromatogram (TIC) of the trimethylsilyl ethers (TMS) of the chemical standards including 25H-5,6α-EC (**di-TMS**) (I), 25H-5,6β-EC (**di-TMS**) (II), 25H-OCDO (**tri-TMS**) (III) and 25H-CT (**tri-TMS, 5-OH**) (IV) in full scan mode in the range of 50–750 m/z.


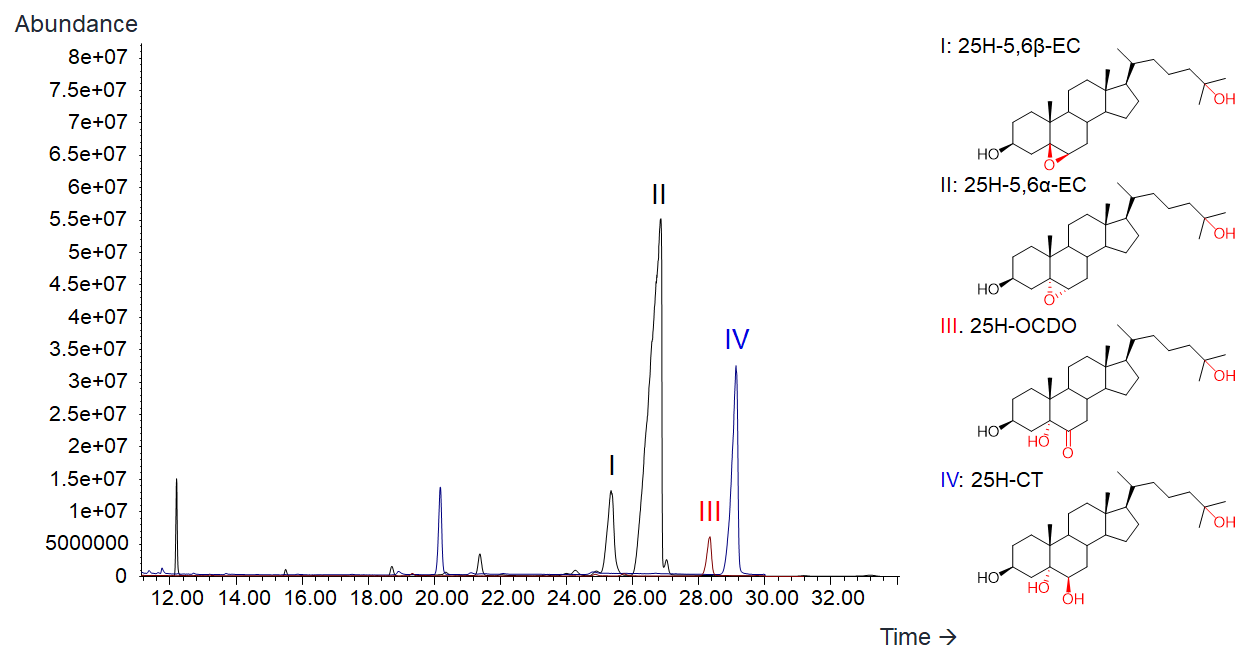


**Supplementary Figure S6:** Mass spectrum of compound I corresponding to the TMS derivative of 25H-5,6ß-EC at the average retention time of 25.11 to 25.47 min.


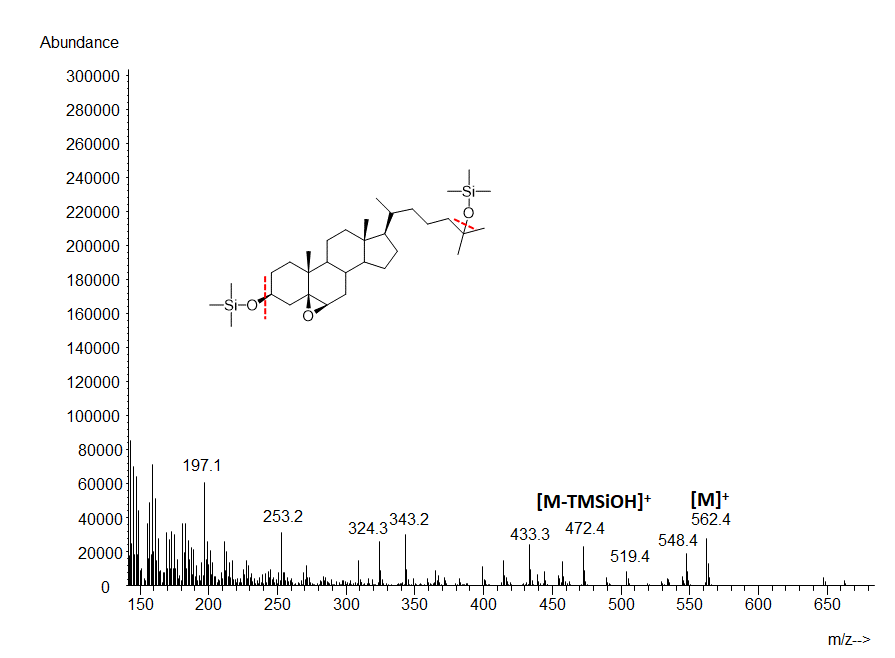


**Supplementary Figure S7:** Mass spectrum of compound II corresponding to the TMS derivative of 25H-5,6α-EC at the average retention time of 26.13 to 26.83 min.


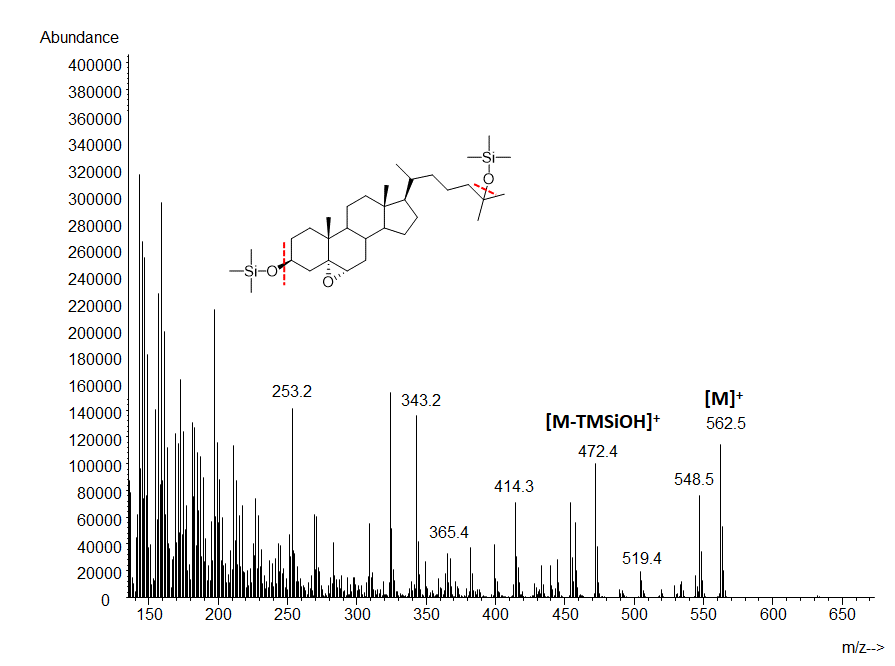


**Supplementary Figure S8:** Mass spectrum of compound III corresponding to the TMS derivative of 25H-OCDO at the average retention time of 28.06 to 28.46 min.


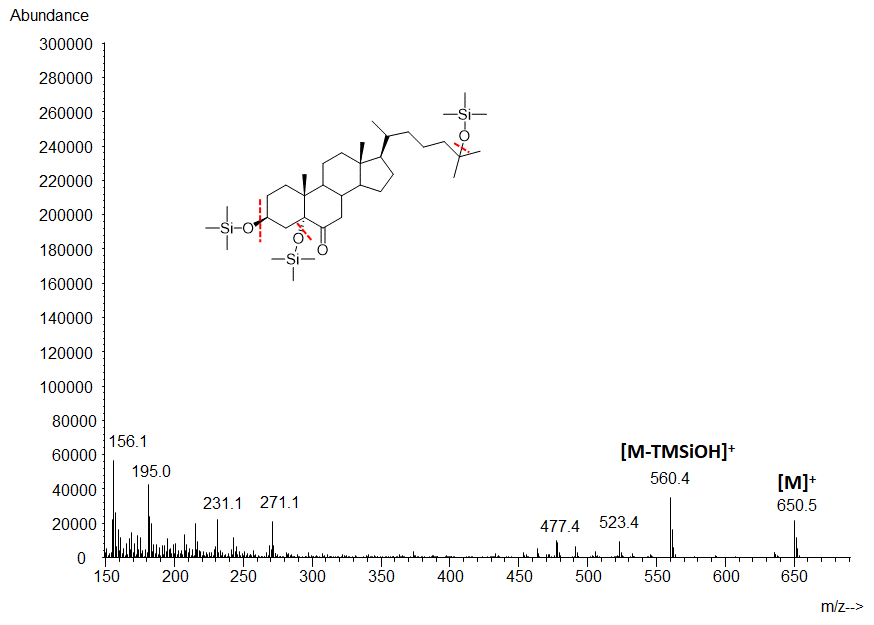


**Supplementary Figure S9:** Mass spectrum of compound IV corresponding to the TMS derivative of 25H-CT at the average retention time of 28.83 to 29.21 min.


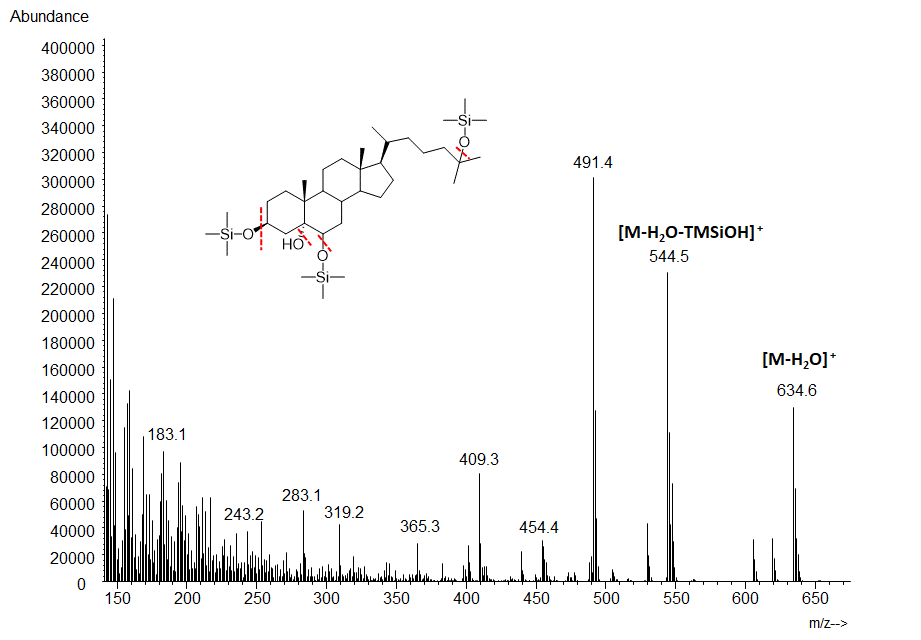


**Supplementary Table 2:**

mRNA expression of bile acid biosynthesis enzymes in cell lines used in this study. Data are taken from the Human Protein Atlas (<https://www.proteinatlas.org/>). TPM: transcripts per kilobase million.

| mRNA (TPM) |  | MCF7 | MDA-231 | MDA-468 | HepG2 |
| --- | --- | --- | --- | --- | --- |
| CYP27A1 | Sterol 27-hydroxylase | 0.0 | 5.1 | 39.4 | 217.7 |
| CYP7B1 | Sterol 7α-hydroxylase | 0.0 | 0.0 | 0.6 | 0.1 |
| CYP8B1 | sterol 12α-hydroxylase | 0.1 | 0.0 | 1.2 | 2.5 |
| AKR1D1 | aldo-keto-reductase family 1 member D1 | 0.0 | 0.0 | 0.0 | 13.9 |
| AKR1C4 | aldo-keto-reductase family 1 member C4 | 0.0 | 0.0 | 0.0 | 0.7 |
| SLC27A5 | solute carrier family 27 | 24.6 | 7.4 | 21.4 | 35.4 |
| AMACR | α-methylacyl-CoA racemase | 14.3 | 14.8 | 6 | 21.8 |
| ACOX2 | acyl-coA oxidase 2 | 0.0 | 0.1 | 0.0 | 15.8 |
| HSD17B4 | hydroxysteroid 17β dehydrogenase 4 | 108.1 | 88.1 | 92.3 | 149.9 |
| SCP2 | sterol carrier protein 2 | 77.4 | 123.8 | 116.0 | 115.5 |
| BAAT | bile acid-CoA:amino acid N-acyltransferase | 0.1 | 0.1 | 0.0 | 2.2 |
| ACOT8 | acyl-CoA thioesterase 8 | 30.1 | 29.9 | 21.7 | 24.7 |
